# Caregiving at end‐of‐life: How do family structure and dementia status impact antidepressant and anxiolytic prescriptions among families?

**DOI:** 10.1002/alz.14590

**Published:** 2025-05-30

**Authors:** Eli Iacob, Mike Hollingshaus, Rebecca L. Utz, Djin L. Tay, Katherine A. Ornstein, Rachael Alexander, Pamela Barrientos, Lee Ellington, Mike Newman, Tom Belnap, Amy M. Cizik, Ken R. Smith, Huong D. Meeks, Caroline E. Stephens

**Affiliations:** ^1^ College of Nursing University of Utah Salt Lake City Utah USA; ^2^ Kem C. Gardner Policy Institute University of Utah Salt Lake City Utah USA; ^3^ Sociology Department University of Utah Salt Lake City Utah USA; ^4^ School of Nursing Johns Hopkins University Baltimore Maryland USA; ^5^ University of Utah Health Sciences Center Salt Lake City Utah USA; ^6^ Healthcare Delivery Institute Intermountain Healthcare Salt Lake City Utah USA; ^7^ Department of Orthopedics University of Utah Salt Lake City Utah USA; ^8^ Huntsman Cancer Institute University of Utah Salt Lake City Utah USA; ^9^ Department of Pediatrics University of Utah Salt Lake City Utah USA

**Keywords:** antidepressant, anxiolytic, caregiving, dementia, family, mental health, Utah population database

## Abstract

**INTRODUCTION:**

End‐of‐life (EOL) caregiving is associated with stress and burden, especially for persons with dementia. Little is known, however, about how dementia diagnosis, family structure, and co‐residence influence the prevalence of antidepressants and anxiolytics (psychiatric prescriptions) among spouses and adult children during EOL caregiving.

**METHODS:**

This was a retrospective cohort study of spouses (*n* = 82,321) and adult children (*n* = 367,888) linked to decedents with (*n* = 35,482) and without dementia (*n* = 121,548) between 1998 and 2016. Multivariable logistic regression analyses examined differences in prescription rates by decedent dementia status and family characteristics.

**RESULTS:**

Decedents’ dementia status was associated with increased odds of psychiatric prescription for all family members (wives: odds ratio [OR] = 1.22 [1.13–1.31]; husbands: OR = 1.15 [1.00–1.32]; sons: OR = 1.01 [1.01–1.14]; daughters: OR = 1.05 [1.01–1.10]). Co‐residence with the decedent reduced odds of prescriptions for daughters (OR = 0.78 [0.72–0.84]) and sons (OR = 0.73 [0.66–0.81]).

**DISCUSSION:**

Dementia status, family structure, and co‐residence may serve as useful indicators for assessing psychosocial needs, allowing health‐care professionals to better identify and support families during EOL caregiving.

**Highlights:**

- Antidepressant and anxiolytic prescriptions may be influenced by the availability, proximity, and structure of family associated with persons with dementia; therefore, assessing family dynamics is essential in holistic dementia care.
- Family caregivers of individuals with dementia were more likely than non‐dementia caregivers to have a psychiatric prescription in the year leading up to the decedent's death. Findings underscore the added stress of dementia end‐of‐life (EOL) care and the need for greater support during this critical pre‐death period.
- Families in which children co‐resided with the decedent and those with more available members generally had lower odds of receiving psychiatric prescriptions. This suggests that family structure, living arrangements, and availability may serve as useful indicators for assessing psychosocial needs, allowing health‐care professionals to better identify and support families requiring assistance during EOL dementia caregiving.

## 1 BACKGROUND

Nearly 18 million unpaid family caregivers play a critical role in the care of older adults as they navigate chronic illness, disability, and end of life (EOL).^1^ Spouses are the most common caregivers, with adult children the next most likely family to provide EOL care.^2^ Family caregivers support the health and well‐being of the aging population by helping with activities of daily living, transportation, finance management, and facilitation of medical needs providing > 36 billion hours of unpaid care with an economic value roughly equating $600 billion.^3^ Unfortunately, these family caregivers often pay an additional price in terms of their own physical and mental health.^4^

Compared to the general population, caregivers report greater stress, burden, anxiety, and depression.^5^ These symptoms are associated with mental health use resulting in prescriptions of anxiolytics and antidepressants.^6^ In the United States, 6.5 million people live with Alzheimer's D = disease or related dementias (hereafter referred to as dementia)—a number projected to double by 2060.^7^ Dementia caregivers spend significantly more time caregiving compared to other illnesses (92 vs. 65 hours per month).^8^ Studies estimate the prevalence of depression and anxiety in dementia caregivers to be as high as 30% to 40%.^9^, ^10^, ^11^, ^12^, ^13^, ^14^, ^15^ A registry‐based study of > 41,000 widows in Finland identified that antidepressant use among dementia caregivers was significantly higher than non‐dementia caregivers 3 to 4 years prior to care‐recipient death.^16^

Risk of caregiver depressive symptomology is associated with characteristics of being White, having a lower education level, living in an urban area, co‐residence, and disease severity.^17^, ^18^, ^19^, ^20^, ^21^ While primary caregivers often receive support from other family, the emotional toll of caregiving remains worse for female caregivers.^22^ Female caregivers often take on greater responsibilities and spend more time caregiving than their male counterparts, especially during EOL care.^11^ Population studies show more female caregivers (28.5%) are prescribed antidepressants compared to males (21.1%).^23^ New antidepressant use significantly increases among spouse/partner caregivers before and after the care recipient's death, but not in adult children.^24^ These patterns are similar for dementia caregivers as well.^16^

Caregiver well‐being is influenced by the availability of family support, family functioning, conflict, and feelings of isolation, all of which are linked to increased depression, distress, and burden.^25^, ^26^, ^27^ While the intersections of sex and relationship to care recipient are well‐known contributors to antidepressant use, less is known from a population perspective about the availability of close family networks that may buffer the emotional impact of dementia caregiving. Prior epidemiological research has used large population‐based datasets to investigate key questions about family caregiving and caregiver well‐being,^28^ including how caregiver stress varies across different family configurations and caregiving roles. Prior studies have not used detailed, population‐based analyses to examine the association between family structure, dementia status, and prevalence of psychiatric prescriptions among caregivers during the EOL period. Understanding how dementia EOL care impacts, and is potentially shared within, diverse family structures can help inform more targeted dementia caregiver interventions.

Using the only available family‐linked EOL caregiving population‐based dataset in the United States, this study examined prevalence of antidepressants and anxiolytics (hereafter referred to as psychiatric medications or prescriptions) in dementia and non‐dementia caregivers throughout the year prior to decedent's death. Consistent with other studies,^29^, ^30^ this study posited that the use of these psychiatric medications is a proxy for clinically relevant caregiver stress. We hypothesized that psychiatric prescriptions would be more likely among those who: (1) are caring for persons with dementia, (2) co‐reside with the person requiring care, and (3) have smaller families including fewer daughters/sons that could contribute to the shared responsibilities of caregiving. Given the gendered nature of caregiving, mental health morbidity, and family relationships,^31^ we estimated the risk for psychiatric prescription separately for sons, daughters, husbands, and wives. Understanding gained from this study will directly respond to the urgent calls from the National Academies of Science and Medicine and the Recognize, Assist, Include, Support, & Engage (RAISE) Family Caregiving Advisory Council^1^, ^2^ to help inform and support the development of programs that prioritize the health and well‐being of family caregivers.^32^

## 2 METHODS

### 2.1 Data source

The Utah Population Database (UPDB) links vital records (birth certificates, death certificates, marriage certificates, etc.); statewide medical records from the Utah Department of Health including surgery, emergency department visits, payer claims data, Medicare and Medicaid services; and detailed medical and pharmacy records from the two largest health‐care systems (U Health and Intermountain Health), which cover > 90% of the state's total medical care.^33^ Importantly, this data resource allows linkage of individuals with their first‐degree family members including spouses, parents, and children. The Utah Caregiving Population Science (C‐PopS) cohort^34^, ^35^, ^36^, ^37^, ^38^ drawn from the larger UPDB has been previously used to answer specific research questions about EOL caregiving.

**RESEARCH IN CONTEXT**

1. **Systematic review**: Authors reviewed all recent family dementia caregiving literature using traditional (e.g., PubMed) sources applicable to this study; relevant citations are appropriately cited. Prior epidemiological research has used large population‐based datasets to investigate family caregiving and caregiver well‐being, including how caregiver stress varies across different family configurations, caregiving roles, and dementia.
2. **Interpretation**: This study is the first population‐based examination of how dementia diagnosis, family structure, and living arrangements may influence the prevalence of antidepressant and anxiolytic prescriptions among spouses and adult children highlighting their mental health burden during the EOL caregiving period. Addressing the mental health needs of dementia caregivers may have significant implications for healthcare policy and is consistent with our review of the current literature.
3. **Future directions**: Exploration of positive or negative impact of family relationships on mental health of EOL dementia caregivers can inform targeted interventions to alleviate caregiver stress which aligns with national priorities.

### 2.2 Sample

**FIGURE 1:**
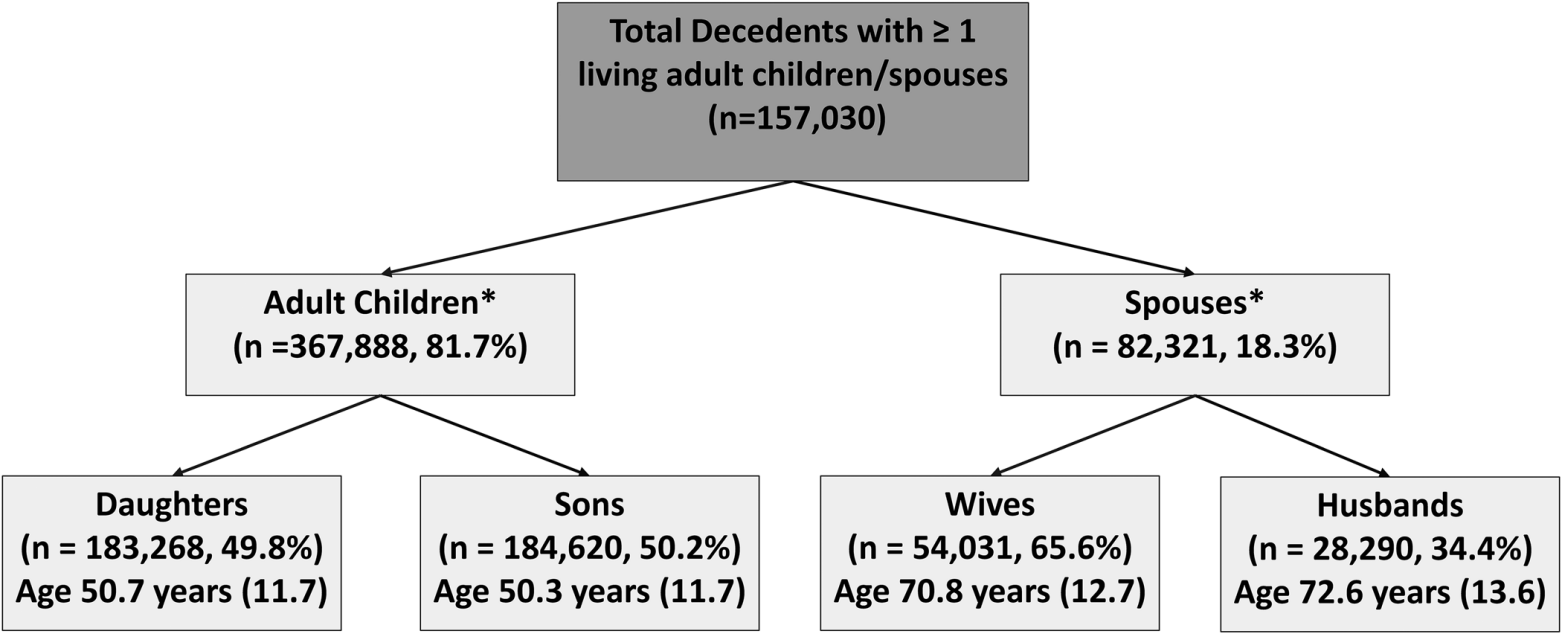
Number of records from overall datasource by relationship type and sex from the Utah C‐PopS datasource. % represent proportion of parent box. *Figure shows number of family‐decedent pairs. A single family can have multiple decedents and thus may appear in the figure more than once. In this sample, *n* = 103,769 (35.0 %) of adult children and *n* = 31,455 (38.2%) of spouses had more than one decedent.

The C‐PopS cohort comprises all decedents over the age of 20 who died of natural causes in the state of Utah between 1998 and 2016. Each decedent was linked to their first‐degree family members (any living spouse, child, siblings, and parent) who was at least ≥ 20 years at time of decedents’ death and living in Utah around the time of the death. This retrospective cohort study examined spouses and adult children of decedents with and without a diagnosis of dementia. Our unit of statistical analysis was a family–decedent pair. Given the structure of the dataset, family can have multiple decedents, decedents can have multiple family, and an individual can serve as both a family to a decedent and later as a decedent themselves. As shown in Figure 1, our final analytic sample consisted of *n* = 82,321 unique spouse–decedent pairs (54,031 wives and 28,290 husbands) and *n* = 367,888 child–decedent pairs (183,268 daughters and 184,620 sons) with *n* = 103,769 (35.0%) adult children and *n* = 31,455 (51.3%) spouses having more than one decedent. This study was approved by the University of Utah Institutional Review Board (IRB) #0017703 and Utah Resource for Genetics and Epidemiology (RGE) #00005823.

### 2.3 Variables

#### 2.3.1 Dependent variable

The dependent variable was prescription of any antidepressant and/or anxiolytic for the family in the 1 year preceding the decedent's death (see Table S1 in supporting information for list of medications) coded as a simple dummy variable (1 = any prescription, 0 = no prescription). Prescriptions were based on medication orders put into the electronic medical record (EMR) within inpatient, outpatient, emergency department, and primary care settings. Though off‐label use of some psychiatric medications may take place^39^ (e.g., for pain), we posit that antidepressants and anxiolytics are commonly used for depression and anxiety and therefore represent good proxies for potential stress and burden felt by the family during the EOL caregiving period.^30^, ^40^

#### 2.3.2 Key exposure variables

Key independent variables included dementia status of the decedent and specific family characteristics describing the family–decedent unit of analysis. Dementia status was based on the following International Classification of Diseases (ICD)‐9 codes (290x, 294.1, 331.1x, 331.2x, 331.82x) and ICD‐10 codes (F00x, F01x, F02x, F03x, F05.1x, G30x, G31.0x, G31.1x) found in EMRs of the decedent during the 2 years prior to death. Given that dementia is widely underreported as a cause of death,^41^ it is common practice to define dementia‐related deaths from medical records rather than solely from a death record. A dummy variable was used to indicate whether family and decedent were believed to live in the same household at the time of decedent death. Family size was defined as a categorical variable including 0, 1, 2, or 3+ levels to describe the presence and number of sons, daughters, brothers, sisters associated with each family–decedent pair. Of note, because these are blood relations based on the decedent for the family they could be either full‐ or half‐siblings. A continuous measure to control for the number of parents and siblings of the decedent was also included. Finally, child models had an indicator for presence of a decedent spouse.

#### 2.3.3 Decedent covariates

Models controlled for year of death to account for potential change in prescription rates in the population between 1998 and 2016,^42^ decedent age, and place of death (indicator with home, hospital, other/assisted living/hospice facility, or unknown as categories). The Charlson Comorbidity Index (CCI),^43^ an index of 19 serious chronic conditions based on diagnosis codes, was used to assess family levels of comorbidity. Higher numbers indicate poorer heath and were categorized as 0, 1 to 2, 3 to 4, 5+, or unknown.

#### 2.3.4 Family covariates

Covariates were based on individual family members’ characteristics at the time of the decedent's death. These included the family member's age; highest level of education attained (categorized as high school, some college, college or beyond, or missing); and a binary indicator for whether the family member was born in Utah, used as a proxy for general family availability.^34^ Baseline health of family members was assessed using the CCI, measured 12 to 24 months prior to the decedent's death. Race/ethnicity was categorized as non‐Hispanic White, non‐Hispanic non‐White, and Hispanic, with data availability limiting finer granularity. County type was classified as urban, frontier, rural, or unknown. Marital status of children at the time of the decedent's death was categorized as married, divorced/separated, widowed, single/other, or missing.

### 2.4 Statistical analysis

Descriptive statistics, chi‐square tests and Student *t* tests were performed to describe decedent cohort and their family, and to compare sociodemographic, socioeconomic, and death characteristics. Demographic and clinical characteristics of entire sample and prevalence of psychiatric prescriptions 1 year prior to decedent death were stratified by relation type and sex (husband, wife, son, daughter) and by whether the decedent had a diagnosis of dementia or not. Consistent with prior research, multivariable logistic regression models were run separately for wives, husbands, sons, and daughters.^24^, ^34^ Because families often consist of interdependent members, we calculated robust standard errors using the sandwich estimator^44^ clustered around each decedent. For all models, we report odds ratios (ORs) with 95% confidence intervals (CIs) and significance levels (*P*), using two‐sided tests with alpha set at *P* < 0.05. All data processing and analyses were conducted in the R environment statistical program R version 4.0.5 (2021‐03‐31) with the glmr^45^ and lmtest^46^ packages.

## 3 RESULTS

### 3.1 Sample description

Of the decedents, 35,482 (22.6%) had a diagnosis of dementia. Among family members, 23.9% of daughters, 23.6% of sons, 17.9% of wives, and 17.6% of husbands had a decedent with dementia. More than 70% of decedents had children, and among them, 54.1% of daughters and 54.7% of sons had a decedent parent with a surviving spouse. Nearly all spouses co‐resided (99.3% wives, 99.0% husbands), while only 5.9% of daughters and 6.6% of sons lived with the decedent. Co‐residence rates varied by dementia status for sons (7.0% without dementia vs. 5.6% with dementia, *P* < 0.001), but not for daughters (5.9% without dementia vs. 6.0% with dementia, *P* = 0.327). (See Table 1 for further details.)

**TABLE 1:** Demographic and clinical characteristics of family separated by relation and sex.

	Children of decedents		Spouses of decedents	
	Daughters	Sons	Wives	Husbands
	(*n* = 183,268)	(*n* = 184,620)	(*n* = 54,031)	(*n* = 28,290)
	*n* (%)	*n* (%)	*n* (%)	*n* (%)
*Key exposure variables*				
Dementia status				
Yes	43,877 (23.94)	43,504 (23.56)	9677 (17.91)	4984 (17.62)
Coresident with decedent				
Yes	10,807 (5.90)	12,245 (6.63)	53,675 (99.34)	28,002 (98.98)
Decedent daughters				
0	0 (0.00)	49,777 (26.96)	23,053 (42.67)	12,777 (45.16)
1	51,624 (28.17)	63,959 (34.64)	14,770 (27.34)	7561 (26.73)
2	62,135 (33.90)	41,957 (22.73)	9608 (17.78)	4803 (16.98)
3+	69,509 (37.93)	28,927 (15.67)	6600 (12.22)	3149 (11.13)
Decedent sons				
0	49,816 (27.18)	0 (0.00)	22,806 (42.21)	12,493 (44.16)
1	62,390 (34.04)	50,611 (27.41)	14,580 (26.98)	7621 (26.94)
2	40,911 (22.32)	61,114 (33.10)	9596 (17.76)	4828 (17.07)
3+	30,151 (16.45)	72,895 (39.48)	7049 (13.05)	3348 (11.83)
Decedent any child				
Yes	183,268 (100.00)	184,620 (100.00)	39,183 (72.52)	19,977 (70.62)
Decedent spouse				
Yes	99,229 (54.14)	100,978 (54.70)	54,031 (100.00)	28,290 (100.00)
*Decedent covariates*				
Decedent age (mean, SD)	79.62 (11.13)	79.40 (11.18)	74.84 (12.57)	70.81 (13.58)
Decedent sex (%)				
Female	94,702 (51.67)	94,660 (51.27)	8 (0.01)	28,276 (99.95)
Insurance status (%)				
Medicaid only	3354 (1.83)	3383 (1.83)	1051 (1.95)	1005 (3.55)
Medicare and Medicaid	9845 (5.37)	9882 (5.35)	1509 (2.79)	936 (3.31)
Medicare only	116,962 (63.82)	117,808 (63.81)	34,597 (64.03)	16,811 (59.42)
Neither	53,107 (28.98)	53,547 (29.00)	16,874 (31.23)	9538 (33.72)
Location of death (%)				
Residence	75,574 (41.24)	74,163 (40.17)	23,521 (43.53)	12,639 (44.68)
Hospital	54,589 (29.79)	55,866 (30.26)	19,748 (36.55)	10,313 (36.45)
Other*	48,583 (26.51)	50,113 (27.14)	9543 (17.66)	4682 (16.55)
Unknown/missing	4522 (2.47)	4478 (2.43)	1219 (2.26)	656 (2.32)
Cause of death (%)				
Cancer	37,416 (20.42)	37,930 (20.54)	14,815 (27.42)	8812 (31.15)
COPD	8367 (4.57)	8314 (4.50)	2669 (4.94)	987 (3.49)
CVD	12,781 (6.97)	12,990 (7.04)	2979 (5.51)	1893 (6.69)
Dementia	16,528 (9.02)	16,379 (8.87)	3116 (5.77)	2125 (7.51)
Heart	47,040 (25.67)	47,460 (25.71)	13,619 (25.21)	5120 (18.10)
Others	61,136 (33.36)	61,547 (33.34)	16,833 (31.15)	9353 (33.06)
Comorbidity index (%)				
0	21,152 (11.54)	21,416 (11.60)	5519 (10.21)	2982 (10.54)
1 to 2	60,016 (32.75)	60,517 (32.78)	15,937 (29.50)	8901 (31.46)
3 to 4	44,663 (24.37)	45,257 (24.51)	14,317 (26.50)	7823 (27.65)
5+	35,388 (19.31)	35,527 (19.24)	13,489 (24.97)	6319 (22.34)
Unknown	22,049 (12.03)	21,903 (11.86)	4769 (8.83)	2265 (8.01)
*Family covariates*				
Age (time of decedent death)	50.65 (11.66)	50.33 (11.66)	70.79 (12.69)	72.57 (13.64)
Born in Utah (%)	169,366 (92.41)	171,221 (92.74)	33,441 (61.89)	17,040 (60.23)
Marital status				
Married	127,751 (69.71)	133,746 (72.44)		
Divorced	21,305 (11.63)	18,524 (10.03)		
Others	26,491 (14.45)	30,046 (16.27)		
Widowed	7721 (4.21)	2304 (1.25)		
Race/ethnicity				
White non‐Hispanic	169,811 (92.66)	171,541 (92.92)	50,137 (92.79)	26,205 (92.63)
Hispanic	9167 (5.00)	8130 (4.40)	2353 (4.35)	1246 (4.40)
Non‐White non‐Hispanic	4290 (2.34)	4949 (2.68)	1541 (2.85)	839 (2.97)
Comorbidity Index (%)				
0	5985 (3.27)	7157 (3.88)	8148 (15.08)	4196 (14.83)
1 to 2	4213 (2.30)	5041 (2.73)	7762 (14.37)	4984 (17.62)
3 to 4	1543 (0.84)	1773 (0.96)	2717 (5.03)	2318 (8.19)
5+	825 (0.45)	1129 (0.61)	1310 (2.42)	1301 (4.60)
Unknown	170,702 (93.14)	169,520 (91.82)	34,094 (63.10)	15,491 (54.76)
Education (maximum)				
Less than HS	11,310 (6.17)	8172 (4.43)	9936 (18.39),	4668 (16.50)
HS grad	55,767 (30.43)	45,413 (24.60)	16,482 (30.50)	7286 (25.75)
Some to post college	90,784 (49.54)	105,968 (57.40)	15,856 (29.35)	12,066 (42.65)
Missing	25,407 (13.86)	25,067 (13.58)	11,757 (21.76)	4270 (15.09)
County type				
Urban	142,585 (77.80)	142,257 (77.05)	40,123 (74.26)	21,108 (74.61)
Frontier	7657 (4.18)	8205 (4.44)	2609 (4.83)	1311 (4.63)
Rural	29,796 (16.26)	30,728 (16.64)	10,730 (19.86)	5574 (19.70)
Unknown	3230 (1.76)	3430 (1.86)	569 (1.05)	297 (1.05)

Abbreviations: COPD, chronic obstructive pulmonary disorder; CVD, cerebrovascular disease; HS, high school.

#### 3.1.1 Prevalence of antidepressant or anxiolytic prescriptions

Wives (10.2%) were more likely than husbands (5.8%), and daughters (7.9%) were more likely than sons (4.1%), to have a psychiatric prescription in the year prior to decedent death (see Table 2). Medication prevalence was consistently higher among family members of individuals with dementia compared to those without dementia. Specifically, 13.4% of wives, 7.4% of husbands, 9.1% of daughters, and 4.9% of sons of decedents with dementia received a psychiatric prescription, compared to 9.5%, 5.5%, 7.6%, and 3.9%, respectively, in families without dementia.

**TABLE 2:** Prevalence of spouse/adult child prescription of antidepressants or anxiolytics 1 year prior to decedent death by decedent dementia status and family sex and relation.

	Total	%	No dementia	%	Dementia	%
Daughters						
No Rx	168,722	92.1%	128,822	92.4%	39,900	90.9%
Rx	14,546	7.9%	10,569	7.6%	3977	9.1%
Sons						
No Rx	177,053	95.9%	135,665	96.1%	41,388	95.1%
Rx	7567	4.1%	5451	3.9%	2116	4.9%
Wives						
No Rx	48,528	89.8%	40,144	90.5%	8384	86.6%
Rx	5503	10.2%	4210	9.5%	1293	13.4%
Husbands						
No Rx	26,645	94.2%	22,028	94.5%	4617	92.6%
Rx	1645	5.8%	1278	5.5%	367	7.4%
Total						
No Rx	420,948	93.5%	326,659	93.8%	94,289	92.4%
Rx	29,261	6.5%	21,508	6.2%	7753	7.6%

Abbreviation: Rx, prescription.

#### 3.1.2 Regression models

**FIGURE 2:**
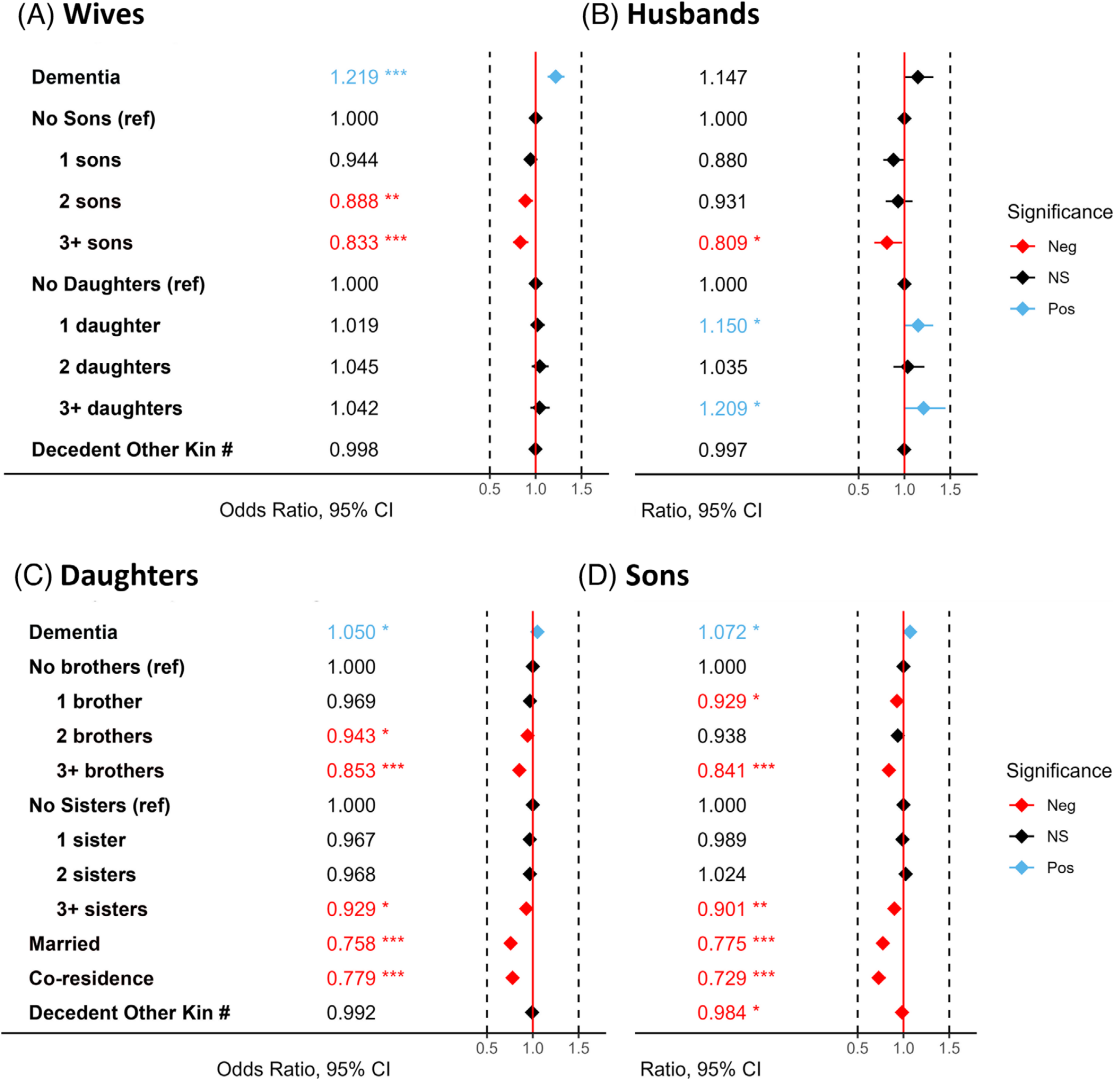
Odds ratios are shown for mainpredictors of interest with significant increased odds shown in blue, decreasedodds in red, and non‐significant odds in grey for A) wives B) Husbands C)Daughters and D) Sons. All models simultaneously adjusted for decedent year ofdeath, decedent age, location of death, decedent CCI, and decedent sex (inchild models). Family covariates not shown include age, ethnicity, born inUtah, CCI, education, and urbanicity. *p < 0.05; **p < 0.01; ***p < 0.001. CI, confidence interval; Neg, negative; NS, not significant; Pos, positive.

As shown in Figure 2, decedent diagnosis of dementia was associated with 22% increased odds of psychiatric prescriptions for wives (OR = 1.22 [95% CI 1.13, 1.32]), 5% for daughters (1.050 [1.01, 1.11]), 7% for sons (1.072 [1.01, 1.14]), and 15% trend for husbands (1.15 [1.00, 1.32]).

Compared to having no sons, wives with 2 or 3+ sons had 11% and 17% lower odds of psychiatric prescription (0.89 [0.81, 0.97]) and (0.83 [0.75, 0.92]), respectively. There was no association of number of daughters with psychiatric prescriptions. Compared to having no sons, husbands with 3+ sons had 19% lower odds of psychiatric prescription (0.81 [0.67, 0.98]). Conversely, compared to no daughters, husbands had 15% and 20% higher odds for a single daughter or 3+ daughters (1.15 [1.01, 1.31]) and (1.21 [1.01, 1.45]), respectively. Among daughters, compared to no siblings, there were 6% decreased odds for prescriptions in the presence of 2 brothers (0.94 [0.90, 0.99]), 15% for 3+ brothers (0.85 [0.81, 0.90]), and 7% for 3+ sisters (0.93 [0.88, 0.98]). Similarly, among sons, compared to no siblings, there were 7% decreased odds for psychiatric prescriptions in the presence of 1 brother (0.93 [0.88, 0.99]), 16% for 3+ brothers (0.84 [0.78, 0.91]), and 10% for 3+ sisters (0.90 [0.83, 0.97]).

Compared to unmarried children, there was significantly decreased odds of prescriptions in married daughters: 24% (0.76 [0.73, 0.79]) and married sons: 23% (0.78 [0.73, 0.82]). Finally, co‐residing with the decedent was also associated with decreased odds in both daughters 22% (0.78 [0.72, 0.84]) and sons 27% (0.73 [0.66, 0.81]). Tables S 2 and S 3 in supporting information summarize the full logistic results including the estimated ORs associated with all covariates.

## 4 DISCUSSION

This is the first US study to examine the association of a dementia diagnosis and family structure on psychiatric prescriptions among family members in the year preceding the death of their spouse or parent. Results revealed that decedent dementia status was associated with increased odds of psychiatric prescription for all family members, with key differences by family size, structure, and characteristics. Residing with the decedent was associated with lower odds of psychiatric prescriptions among children. Both wives and husbands were less likely to have psychiatric prescriptions if they had sons; while husbands, but not wives, had higher odds if daughters were present. Additionally, the presence of siblings reduced the likelihood of psychiatric prescriptions for sons and daughters. Improving our understanding of how dementia EOL care impacts, and is potentially shared within, diverse family structures provides vital information that may improve how best to design and deliver dementia caregiver interventions.

### 4.1 Role of dementia diagnosis

Dementia caregiving is longer in duration and more intensive, compared to caring for an older adult with other chronic illnesses.^7^, ^11^ Thus, it is not surprising that we found that prevalence of psychiatric prescriptions was consistently higher among family members of individuals with dementia compared to those without dementia. The prolonged caregiving trajectory associated with dementia, coupled with the progressive nature of the disease, likely leads to chronic stress and caregiver burden, further contributing to the mental health distress commonly observed among family members serving as dementia EOL caregivers.^14^, ^15^, ^16^, ^40^, ^47^ Study findings may reflect increased dementia EOL burden and/or self‐care behaviors related to mood (e.g., seeking care for needed mental health support). In both circumstances, findings highlight the need for targeted mental health support and interventions for dementia caregivers, particularly in the EOL stage when the burden may be most acute.^40^, ^47^ Providing adequate support and resources to these caregivers is crucial, not only to improve their well‐being but also to ensure they can continue to provide high‐quality care.

### 4.2 Role of family characteristics

While we hypothesized that spouses who had more children would have decreased odds of psychiatric prescriptions, our findings were more nuanced. For example, compared to being an only child, children with more siblings had decreased odds of prescriptions. Perhaps having more siblings promotes greater sharing of caregiving responsibilities, reduces social isolation, and offsets financial strain—all factors that may impact caregiver mental well‐being, particularly among adult children who are providing EOL care to their parents. Of note, not only the number but the quality of family dynamics is important to consider, with prior research suggesting that more stressful or non‐supportive family relationships are most likely related to increased risk of depression.^25^, ^26^, ^27^ If one child is more involved in caregiving than another, the presence of other siblings who are not helping could lead to increased perceived caregiver burden^26^ and therefore could increase the odds of prescriptions. Our dataset, while large, does not permit a more granular examination of the qualitative nature of family dynamics, so future studies should consider the interplay of family size, intra‐family relationship quality, and level of shared caregiver responsibilities among multiple family members.

### 4.3 Impact of co‐residence

A key variable of interest was co‐residence, as family who co‐reside are more likely to be involved in caregiving than those who were not co‐residing. Only 6% of adult children co‐resided with parents, yet for both sons and daughters, co‐residence was found to be protective against receiving psychiatric prescriptions. Co‐residence may be protective and reduce caregiver stress for several reasons. For example, geographic proximity allows for immediate responses to any emergent situations, offering peace of mind that comes from being physically close.^25^, ^48^ This may be particularly important for sons and daughters who are “sandwich generation caregivers” concurrently managing child care needs.^49^, ^50^, ^51^ Such closeness can further foster a deeper understanding of the parent's needs and preferences, making caregiving more intuitive and less uncertain.^27^ Sharing living expenses may also alleviate some financial strain, providing more resources for both the caregiver and decedent.^29^ Additionally, emotional support and familial bonds are strengthened through daily interactions and shared experiences, creating a supportive environment that buffers against the stress of caregiving challenges.^40^ Overall, living together can enhance the quality of care provided while simultaneously reducing the caregiver's stress levels, particularly at EOL.^47^

### 4.4 Clinical and policy implications

Study findings underscore the significant mental health challenges faced by family members caring for individuals with dementia at EOL. Elevated rates of psychiatric prescriptions among these caregivers highlight the need for targeted mental health interventions. Psychosocial interventions, such as psychoeducational programs, have been effective in reducing caregiver burden and improving mental health outcomes.^52^ Family dynamics also play a crucial role in caregiver stress. Supportive family structures can alleviate caregiver burden, while dysfunctional dynamics may exacerbate stress. Interventions that address family functioning, such as psychotherapeutic treatment and caregiver support or respite programs, can reduce caregiver distress.^53^, ^54^ Co‐residence with the care recipient presents unique challenges and benefits. While proximity can enhance caregiving, it may also increase stress due to the constant demands of care.

Addressing the mental health needs of dementia caregivers has significant implications for health‐care policy and could improve cost efficiencies by delaying time to nursing home placement and reducing burdensome care transitions at EOL.^55^ Tele‐health interventions have shown promise in reducing caregiver depression and mental health impairment, offering support without the need for physical presence.^56^ Health‐care providers should assess family structures and living arrangements^57^ when designing support strategies for dementia caregivers. Tailoring interventions to these factors can enhance their effectiveness, ensuring that caregivers receive the appropriate support to manage their mental health during the EOL caregiving journey.

### 4.5 Study limitations

This study has several limitations to consider. Prescription use in the last year of life may not be a sufficient time window to see differences as supported by research that found increased medication prevalence up to 3 to 4 years prior to death in widows but not widowers.^16^ Results may have differed if a longer period prior to death was examined. Our dataset also only contains information on spouses and first‐degree family who live in the state thus excluding domestic partners, stepchildren, siblings, friends, community members, and family members outside of the state who may also play a pivotal role in caregiving and social support.^40^, ^55^ Importantly, the Utah C‐PopS dataset cannot distinguish between caregiving and non‐caregiving family and lacks granular data regarding the caregiving context (e.g., caregiving frequency, nature of caregiving activities), which has been shown to impact caregiver burden and distress.^12^, ^13^

Furthermore, our data only show psychiatric medication patterns and cannot directly measure caregiver mental health or stress. Last, the Utah C‐PopS dataset is restricted to residents of Utah, which may have different family demographics than other states and thus generalizability of findings is unclear. Nonetheless the composition of Utah's demographic is similar to many states in the Mountain West and has trended in the same direction as the national average since 2000.^58^, ^59^ Future studies should aim to compare the predicted probabilities of prescription prevalence in Utah to those from other states to validate these findings further.

Despite these limitations, this Utah C‐PopS cohort drawn from the UPDB allows for a novel investigation of psychiatric prescriptions as a proxy of clinically relevant stress among the husbands, wives, sons, and daughters of persons with and without dementia at EOL. No other population‐based family‐linked EOL caregiving dataset exists in the United States. Being able to match decedents to multiple first‐degree family members allows for exploration of a family's health status, including psychiatric prescriptions. The large sample size permits inclusion of clinical and demographic variables in both decedents and family to approximate population‐level estimates of adjusted prescription odds based on our predictors of interest. This robust dataset provides future avenues to examine sample subsets and explore interaction variables that may explain further for whom, and under what circumstances, there are increased odds of prescriptions. For example, presence of prescriptions in other family members could serve as an overall familial risk factor. While the current analysis does not fully explore the compounding effect of being a family member to multiple decedents, sensitivity analysis selecting just one decedent per family member found that having had a previous decedent did not significantly impact the association of the other predictors with prescriptions.

While the current analysis operationalized prescriptions as a binary indicator requiring only a single prescription, our future work includes obtaining more detailed information on the number, timing, and duration of prescriptions so that we can better delineate the incidence of new prescriptions compared to prevalence of prescriptions from long‐term users. By controlling for prescriptions prior to death, models could then examine prescription prevalence during bereavement and whether prescriptions varied based on whether the decedent died with or without dementia.^12^, ^16^, ^24^

### 4.6 Conclusion and implications

Families of individuals with dementia are more likely to be prescribed psychiatric medications compared to families of those without dementia, highlighting the challenges involved in EOL care for dementia patients. Moreover, family availability, structure, and living arrangements may serve as useful indicators for assessing psychosocial needs. These data allow health‐care professionals to better identify and tailor support to families requiring assistance during EOL dementia caregiving. Health policy efforts should focus on more comprehensive, equitable, and effective support systems for dementia caregivers to improve the clinical and cost outcomes for both the caregivers and the persons for whom they care.^55^

## CONFLICT OF INTEREST STATEMENT

All authors declare that they have no competing interests. Author disclosures are available in the supporting information.

## CONSENT STATEMENT

This is an exempt study and a waiver for informed consent was approved as defined in University of Utah IRB #0017703.

## Supporting information



Supporting Information

Supporting Information
