# Supplementary material for: Caregiving at end‐of‐life: How do family structure and dementia status impact antidepressant and anxiolytic prescriptions among families?
Source: Alzheimers Dement. 2025 May 30;21(6):e14590. doi: 10.1002/alz.14590 (PMC12125481; doi:10.1002/alz.14590)
Supplement: Supplementary file 1 — Supporting Information [file ALZ-21-e14590-s002.docx]

Supplemental Table 1: List of antidepressant and anxiolytic medications

| **Antidepressant** | **Antidepressant** | **Anxiolytic** |
| --- | --- | --- |
| amitriptyline | isocarboxazid | alprazoLam |
| amoxapine | Levomilnacipran HCl | buspirone |
| Brexanolone | maprotiline | chlordiazepoxide |
| buPROPion | Maprotiline HCl | chlordiazePOXIDE HCl |
| bupropion extended release | mirtazapine | clorazepate |
| buPROPion HBr | nefazodone | Clorazepate Dipotassium |
| buPROPion HCl | Nefazodone HCl | diazepam |
| citalopram | nortriptyline | hydrOXYzine |
| Citalopram Hydrobromide | Nortriptyline HCl | hydrOXYzine HCl |
| clomiPRAMine | paroxetine | hydrOXYzine Pamoate |
| clomiPRAMINE HCl | PARoxetine HCl | lorazepam |
| desipramine | PARoxetine Mesylate | LORazepam-Dextrose |
| Desipramine HCl | phenelzine | LORazepam-Sodium Chloride |
| desvenlafaxine | Phenelzine Sulfate | meprobamate |
| Desvenlafaxine Fumarate | protriptyline | oxazepam |
| Desvenlafaxine Succinate | Protriptyline HCl |  |
| Doxepin HCl | selegiline |  |
| doxepin oral | sertraline |  |
| duloxetine | Sertraline HCl |  |
| DULoxetine HCl | tranylcypromine |  |
| escitalopram | Tranylcypromine Sulfate |  |
| Escitalopram &Methfol-B6-B12-D | trazodone |  |
| Escitalopram Oxalate | traZODone HCl |  |
| Esketamine HCl | trimipramine |  |
| fluoxetine | Trimipramine Maleate |  |
| FLUoxetine HCl | venlafaxine |  |
| fluvoxamine | Venlafaxine HCl |  |
| fluvoxaMINE Maleate | vilazodone |  |
| imipramine | Vilazodone HCl |  |
| Imipramine HCl | Vortioxetine HBr |  |
| Imipramine Pamoate |  |  |

Supplemental Table 2: Odds of psychotropic Rx in Wives and Husbands

|  | Wives | | | | Husbands | | | |
| --- | --- | --- | --- | --- | --- | --- | --- | --- |
| Predictors | Odds Ratio | *95% CI* | | *p* | Odds Ratio | *95% CI* | | *p* |
|  |  | *LB* | *UB* |  |  | *LB* | *UB* |  |
| Dementia | **1.219** | 1.130 | 1.315 | **<0.001** | 1.147 | 1.000 | 1.316 | 0.050 |
| *No Sons (ref)* | 1.000 |  |  |  | 1.000 |  |  |  |
| 1 | 0.944 | 0.876 | 1.018 | 0.134 | 0.880 | 0.769 | 1.008 | 0.065 |
| 2 | **0.888** | 0.813 | 0.971 | **0.009** | 0.931 | 0.796 | 1.088 | 0.367 |
| 3+ | **0.833** | 0.753 | 0.921 | **<0.001** | **0.809** | 0.671 | 0.975 | **0.026** |
| *No Daughters (ref)* | **1.000** |  |  |  | 1.000 |  |  |  |
| 1 | 1.019 | 0.944 | 1.100 | 0.631 | **1.150** | 1.006 | 1.314 | **0.041** |
| 2 | 1.045 | 0.957 | 1.142 | 0.325 | 1.035 | 0.880 | 1.218 | 0.676 |
| 3+ | 1.042 | 0.943 | 1.153 | 0.419 | **1.209** | 1.009 | 1.448 | **0.039** |
| Decedent Other Family # | 0.998 | 0.980 | 1.016 | 0.794 | 0.997 | 0.966 | 1.028 | 0.827 |
| Death Year | **1.143** | 1.137 | 1.149 | **<0.001** | **1.144** | 1.133 | 1.156 | **<0.001** |
| Decedent Age | 1.003 | 0.997 | 1.009 | 0.293 | 0.996 | 0.984 | 1.008 | 0.549 |
| *Death at home (ref)* | 1.000 |  |  |  | 1.000 |  |  |  |
| Hospital | 0.918 | 0.857 | 0.983 | **0.015** | 0.985 | 0.875 | 1.110 | 0.808 |
| Other* | **1.215** | 1.119 | 1.318 | **<0.001** | **1.222** | 1.057 | 1.413 | **0.007** |
| Unknown/missing | **1.517** | 1.302 | 1.767 | **<0.001** | 0.993 | 0.735 | 1.343 | 0.965 |
| *Decedent CCI 0 (ref)* | 1.000 |  |  |  | 1.000 |  |  |  |
| Decedent CCI 1-2 | 1.102 | 0.989 | 1.228 | 0.078 | 1.154 | 0.954 | 1.396 | 0.140 |
| Decedent CCI 3-4 | **1.125** | 1.009 | 1.254 | **0.034** | **1.226** | 1.011 | 1.487 | **0.038** |
| Decedent CCI 5+ | **1.150** | 1.031 | 1.282 | **0.012** | 1.211 | 0.995 | 1.476 | 0.057 |
| Decedent CCI unknown | 0.887 | 0.770 | 1.022 | 0.098 | 0.947 | 0.729 | 1.230 | 0.683 |
| Family Age | **0.987** | 0.982 | 0.993 | **<0.001** | **0.988** | 0.976 | 1.000 | **0.046** |
| *Family White non-Hispanic (ref)* | 1.000 |  |  |  | 1.000 |  |  |  |
| Family Hispanic | 0.908 | 0.789 | 1.044 | 0.176 | 0.958 | 0.747 | 1.229 | 0.735 |
| Family Non-Hispanic Non-White | **0.582** | 0.473 | 0.717 | **<0.001** | **0.649** | 0.455 | 0.925 | **0.017** |
| Born in Utah | 1.058 | 0.993 | 1.127 | 0.081 | **1.125** | 1.005 | 1.261 | **0.041** |
| *Family CCI 0 (ref)* | 1.000 |  |  |  | 1.000 |  |  |  |
| Family CCI 1-2 | **1.206** | 1.080 | 1.346 | **0.001** | **1.632** | 1.345 | 1.981 | **<0.001** |
| Family CCI 3-4 | **1.399** | 1.215 | 1.609 | **<0.001** | **1.843** | 1.480 | 2.295 | **<0.001** |
| Family CCI 5+ | **1.709** | 1.443 | 2.024 | **<0.001** | **2.174** | 1.707 | 2.769 | **<0.001** |
| Family CCI unknown | **0.685** | 0.617 | 0.761 | **<0.001** | **0.683** | 0.561 | 0.831 | **<0.001** |
| *Family Less than HS (ref)* | 1.000 |  |  |  | 1.000 |  |  |  |
| Family Hs Grad | **0.872** | 0.798 | 0.952 | **0.002** | 0.959 | 0.808 | 1.137 | 0.631 |
| Family education college/post | **0.870** | 0.794 | 0.952 | **0.003** | 0.997 | 0.849 | 1.171 | 0.969 |
| Family education missing | 1.008 | 0.909 | 1.118 | 0.884 | **1.279** | 1.039 | 1.574 | **0.020** |
| *Family Urban (ref)* | 1.000 |  |  |  | 1.000 |  |  |  |
| Family Frontier | **0.403** | 0.334 | 0.485 | **<0.001** | **0.427** | 0.305 | 0.598 | **<0.001** |
| Family Rural | **0.764** | 0.709 | 0.824 | **<0.001** | **0.875** | 0.768 | 0.996 | **0.043** |
| Family Unknown | **0.743** | 0.553 | 0.999 | **0.049** | 0.762 | 0.460 | 1.263 | 0.291 |

Supplemental Table 3: Odds of psychotropic Rx in Daughters and Sons

|  | Daughters | | | | Sons | | | |
| --- | --- | --- | --- | --- | --- | --- | --- | --- |
| Predictors | Odds Ratio | *95% CI* | | *p* | Odds Ratio | *95% CI* | | *p* |
|  |  | *LB* | *UB* |  |  | *LB* | *UB* |  |
| Dementia | **1.050** | 1.006 | 1.096 | **0.026** | **1.072** | 1.012 | 1.135 | **0.018** |
| *No brothers (ref)* | 1.000 |  |  |  | 1.000 |  |  |  |
| 1 | 0.969 | 0.928 | 1.013 | 0.163 | **0.929** | 0.876 | 0.986 | **0.015** |
| 2 | **0.943** | 0.897 | 0.991 | **0.020** | 0.938 | 0.877 | 1.002 | 0.058 |
| 3+ | **0.853** | 0.806 | 0.903 | **<0.001** | **0.841** | 0.780 | 0.907 | **<0.001** |
| *No Sisters (ref)* | 1.000 |  |  |  | 1.000 |  |  |  |
| 1 | 0.967 | 0.925 | 1.010 | 0.132 | 0.989 | 0.932 | 1.050 | 0.714 |
| 2 | 0.968 | 0.921 | 1.018 | 0.203 | 1.024 | 0.958 | 1.095 | 0.477 |
| 3+ | **0.929** | 0.879 | 0.983 | **0.010** | **0.901** | 0.833 | 0.974 | **0.009** |
| Married | **0.758** | 0.729 | 0.789 | **<0.001** | **0.775** | 0.731 | 0.821 | **<0.001** |
| Co-residence | **0.779** | 0.722 | 0.841 | **<0.001** | **0.729** | 0.659 | 0.807 | **<0.001** |
| Decedent Other Family # | 0.992 | 0.981 | 1.003 | 0.144 | **0.984** | 0.970 | 0.999 | **0.042** |
| Death Year | **1.143** | 1.139 | 1.147 | **<0.001** | **1.151** | 1.146 | 1.156 | **<0.001** |
| Decedent Age | 0.997 | 0.993 | 1.000 | 0.042 | 1.003 | 0.998 | 1.007 | 0.222 |
| *Death at home (ref)* | 1.000 |  |  |  | 1.000 |  |  |  |
| Hospital | 0.986 | 0.943 | 1.030 | 0.526 | 0.980 | 0.923 | 1.040 | 0.500 |
| Other* | **1.062** | 1.015 | 1.111 | **0.009** | 1.045 | 0.984 | 1.111 | 0.154 |
| Unknown/missing | **1.259** | 1.147 | 1.382 | **<0.001** | **1.203** | 1.062 | 1.363 | **0.004** |
| Decedent spouse | **0.942** | 0.906 | 0.979 | **0.002** | 1.002 | 0.950 | 1.056 | 0.951 |
| *Decedent CCI 0 (ref)* | 1.000 |  |  |  | 1.000 |  |  |  |
| Decedent CCI 1-2 | 1.018 | 0.959 | 1.080 | 0.566 | 1.016 | 0.938 | 1.101 | 0.695 |
| Decedent CCI 3-4 | 1.063 | 0.998 | 1.131 | 0.056 | 1.048 | 0.964 | 1.139 | 0.269 |
| Decedent CCI 5+ | **1.102** | 1.033 | 1.176 | **0.003** | 1.054 | 0.966 | 1.150 | 0.234 |
| Decedent CCI unknown | **0.913** | 0.848 | 0.983 | **0.016** | 0.906 | 0.821 | 1.000 | 0.051 |
| Decedent sex = male | 0.974 | 0.937 | 1.012 | 0.174 | **0.936** | 0.889 | 0.985 | **0.012** |
| Family Age | **1.007** | 1.003 | 1.010 | **<0.001** | 0.999 | 0.995 | 1.003 | 0.764 |
| *Family White non-Hispanic (ref)* | 1.000 |  |  |  | 1.000 |  |  |  |
| Family Hispanic | 0.981 | 0.908 | 1.059 | 0.619 | 1.011 | 0.908 | 1.126 | 0.838 |
| Family Non-Hispanic Non-White | **0.644** | 0.563 | 0.736 | **0.000** | **0.615** | 0.516 | 0.733 | **<0.001** |
| Born in Utah | 0.980 | 0.917 | 1.047 | 0.554 | **1.135** | 1.033 | 1.247 | **0.008** |
| *Family CCI 0 (ref)* | 1.000 |  |  |  | 1.000 |  |  |  |
| Family CCI 1-2 | **1.596** | 1.392 | 1.830 | **<0.001** | **1.665** | 1.427 | 1.943 | **<0.001** |
| Family CCI 3-4 | **1.799** | 1.514 | 2.136 | **<0.001** | **2.137** | 1.764 | 2.588 | **<0.001** |
| Family CCI 5+ | **1.817** | 1.468 | 2.249 | **<0.001** | **2.566** | 2.086 | 3.157 | **<0.001** |
| Family CCI unknown | **0.710** | 0.640 | 0.788 | **<0.001** | **0.538** | 0.477 | 0.608 | **<0.001** |
| *Family Less than HS (ref)* | 1.000 |  |  |  | 1.000 |  |  |  |
| Family Hs Grad | **0.926** | 0.859 | 0.997 | **0.042** | 0.927 | 0.824 | 1.043 | 0.208 |
| Family education college/post | **0.799** | 0.743 | 0.861 | **<0.001** | 0.914 | 0.815 | 1.025 | 0.123 |
| Family education missing | **0.820** | 0.753 | 0.893 | **<0.001** | 0.935 | 0.824 | 1.063 | 0.305 |
| *Family Urban (ref)* | 1.000 |  |  |  | 1.000 |  |  |  |
| Family Frontier | **0.312** | 0.272 | 0.358 | **<0.001** | **0.394** | 0.333 | 0.465 | **<0.001** |
| Family Rural | **0.757** | 0.720 | 0.796 | **<0.001** | **0.722** | 0.675 | 0.773 | **<0.001** |
| Family Unknown | **0.405** | 0.346 | 0.473 | **<0.001** | **0.459** | 0.379 | 0.555 | **<0.001** |
